# Evaluation of a Test to Stay Strategy in Transitional Kindergarten Through Grade 12 Schools — Los Angeles County, California, August 16–October 31, 2021

**DOI:** 10.15585/mmwr.mm705152e1

**Published:** 2021-12-31

**Authors:** Kimberly Harris-McCoy, Veronica C. Lee, Cortney Munna, Andrea A. Kim

**Affiliations:** 1 Los Angeles County Department of Public Health, California.

On July 12, 2021, the California Department of Public Health updated COVID-19 school guidance, allowing a Test to Stay (TTS) strategy to increase access to in-person learning* (*1*). The TTS strategy enabled unvaccinated students, exposed in school to a person infected with SARS-CoV-2 (the virus that causes COVID-19), to remain in school while under quarantine, if both the infected person and the exposed person wore masks correctly and consistently throughout the exposure. To stay in school during the quarantine period, the exposed student must remain asymptomatic, wear a mask at school, and undergo twice weekly testing for SARS-CoV-2. To date, few studies have evaluated the impact of TTS on transmission (*2*–*4*). This study evaluated a TTS strategy implemented by Los Angeles County Department of Public Health (LAC DPH). During September 20–October 31, 2021, among 78 school districts, one half permitted TTS; in total, 432 (21%) of 2,067 schools adopted TTS. TTS schools did not experience increases in COVID-19 incidence among students after TTS implementation, and in 20 identified outbreaks in TTS schools,^†^ no tertiary transmission was identified. The ratio of student COVID-19 incidence in TTS districts to that in non-TTS districts was similar before and after TTS adoption (rate ratio = 0.5). Non-TTS schools lost an estimated 92,455 in-person school days during September 20–October 31 while students were in quarantine, compared with no lost days among quarantined students in TTS schools. Non-TTS schools cited resource-related reasons for not adopting TTS; 75% of these schools were in LAC’s most disadvantaged neighborhoods. Preliminary data from LAC suggest that a school-based TTS strategy does not increase school transmission of SARS-CoV-2, and might greatly reduce loss of in-person school days; however, TTS might have barriers to implementation and require resources that are not available for some schools. Continued efforts to simplify school quarantine strategies might help to ensure that all students have access to safe in-person education. Although vaccination remains the leading public health recommendation to protect against COVID-19 for persons aged ≥5 years, schools might consider TTS as an option for allowing students with a school exposure who are not fully vaccinated to remain in the classroom as an alternative to home quarantine.

LAC has 78 public school districts with 2,067 schools for students in transitional kindergarten through grade 12.^§^ Schools require indoor masking, physical distancing where feasible, vaccination, isolation of persons with confirmed cases, contact tracing, quarantining of close contacts, and SARS-CoV-2 testing (*5*). School SARS-CoV-2 testing strategies include weekly testing of asymptomatic, unexposed persons and response testing of persons with symptoms or exposures using SARS-CoV-2 nucleic acid amplification tests or antigen tests. LAC DPH is notified of school COVID-19 cases and close contacts of persons who received positive test results via a secure line list or online survey using REDCap (version 10.3.3; Vanderbilt University).

LAC DPH allowed schools to adopt a TTS strategy starting on September 20, 2021. For asymptomatic, unvaccinated students under quarantine orders,^¶^ TTS was permitted during the quarantine period if the exposure occurred in school and the exposed student and infected person both wore masks correctly and consistently during the exposure. During TTS, contacts could continue in-person academic activities during regular school hours if they remained asymptomatic, wore a mask while at school (indoors, outdoors, and on school buses), received testing twice weekly by a certified testing program or health care provider,** and agreed to quarantine at home when not at school. Contacts could not participate in extracurricular activities or before- or after-school care during the quarantine period.

School COVID-19 cases were defined as a laboratory-confirmed SARS-CoV-2 infection in a person who was at school anytime during the 14 days before their episode date (symptom onset date or the positive SARS-CoV-2 test result date, whichever was earlier). School cases were verified with test results reported by laboratories or health care providers to the LAC DPH Integrated Reporting and Investigation Surveillance System (IRIS). Cases among students with episode dates during August 16–October 31 and school exposures of student close contacts during August 17–October 31 were used to calculate secondary infection risk (number of quarantined contacts with a COVID-19 diagnosis 1–14 days after exposure divided by the total number of quarantined contacts).^††^ COVID-19 student case rates were calculated as the average daily number of student cases during a 7-day period divided by the number of enrolled students.^§§^ COVID-19 student rates are presented with 95% CIs; rates with non-overlapping CIs were considered to be significantly different. COVID-19 student rate ratios were calculated by dividing COVID-19 student case rates in TTS schools by those in non-TTS schools.

School district administrators were interviewed during November 3–16, 2021, to determine whether the district adopted TTS and to assess implementation challenges and reasons for not implementing TTS. TTS districts might have permitted TTS only for certain school levels; therefore, schools were subsequently categorized as having adopted versus not adopted TTS. School outbreak data were reviewed for evidence of tertiary transmission within TTS schools. Tertiary transmission was defined as likely SARS-CoV-2 transmission to a student, from a student participating in TTS who received a positive SARS-CoV-2 test result during the TTS period (i.e., a student with a secondary case). Schools were grouped into quartiles of disadvantage based on the California Healthy Places Index (HPI)^¶¶^ (*6*). Zip codes falling within the lowest HPI quartile represented the most disadvantaged neighborhoods. Among non-TTS schools, estimation of lost in-person school days assumed 5 missed school days for each 7-day student quarantine.*** This analysis was restricted to public school districts; Pasadena Unified School District (USD), Long Beach USD, and non-residents of LAC were excluded.^†††^ SAS statistical software (version 9.4; SAS Institute) was used for all analyses. This public health surveillance activity was reviewed and approved by LAC DPH.

An estimated 1,292,067 LAC public school students returned to school for the 2021–22 academic year, which commenced on August 16, 2021, for most LAC public schools. During August 16–October 31, an average of 462,189 student and staff member SARS-CoV-2 tests were conducted each week in all schools. During the week of August 16, 0.6% of test results were positive, but this percentage declined to 0.2% by October 31 (Figure 1). Among all schools, 12,919 student COVID-19 cases (1% of the student population) and 57,513 student contacts (4% of the student population) were reported during the 10-week observation period; case numbers peaked at 2,270 during the week of August 16, and the number of contacts peaked at 8,589 during the week of August 23.

**FIGURE 1:**
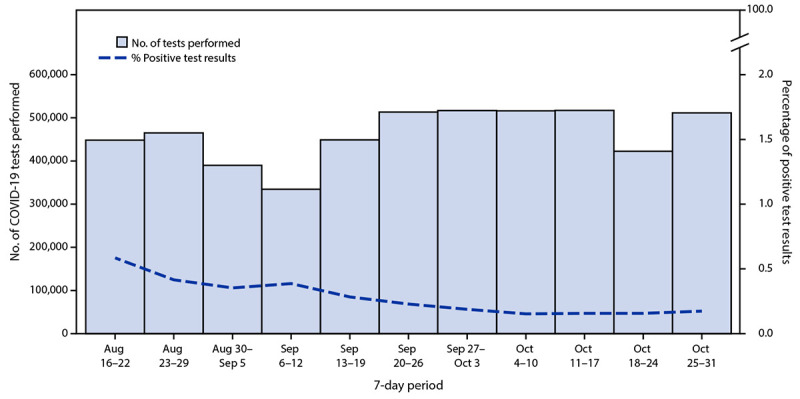
Number of SARS-CoV-2 tests performed and percentage of positive test results* in transitional kindergarten through grade 12 public school districts — Los Angeles County, California, August 16–October 31, 2021 * Weekly data might have included repeat tests for an individual person.

During September 20–October 31, among 78 school districts, 39 (50%) permitted TTS; within these districts, 94% of schools (432 of 452) adopted TTS (Table). These TTS schools constitute 21% of LAC public schools. LAC’s largest school district, which accounts for one third of public school students, did not adopt TTS. Overall, within the 1,635 non-TTS schools, 4,322 COVID-19 cases occurred among 967,188 enrolled students (4.7 cases per 1,000 students); among 18,729 student close contacts, the secondary infection risk was 1.3%. Non-TTS districts lost an estimated 92,455 in-person school days during September 20–October 31 while students were in quarantine. Within the 432 TTS schools, among 324,879 enrolled students, 812 COVID-19 cases occurred (2.5 cases per 1,000 students); among 7,511 student close contacts, the secondary infection risk was 0.7%. As a result of the TTS protocol, no in-person school days were lost among quarantined students participating in TTS. Among 20 school outbreaks that occurred in TTS schools after TTS implementation, three outbreaks included four TTS students who were secondarily infected; contact tracing confirmed seven contacts of these patients and identified no tertiary transmission.

**TABLE:** Characteristics of transitional kindergarten through grade 12 public schools, by school Test to Stay status — Los Angeles County, California, September 20–October 31, 2021

Characteristic	Did not implement TTS	Implemented TTS
**Schools**	**(n = 1,635)**	**(n = 432)**
No. of enrolled students*	967,188^†^	324,879
**Student COVID-19 cases, total**	**4,322**	**812**
Elementary school, no. (% of total)	2,403 (56)	341 (42)
Middle school, no. (% of total)	956 (22)	159 (20)
High school, no. (% of total)	963 (22)	312 (38)
**Student close contacts of COVID-19 cases,^§^ total**	**18,729**	**7,511**
Elementary school, no. (% of total)	9,177 (49)	2,253 (30)
Middle school, no. (% of total)	4,870 (26)	1,878 (25)
High school, no. (% of total)	4,682 (25)	3,380 (45)
Student secondary infection risk^¶^	1.3	0.7
Percentage of schools in the most disadvantaged neighborhoods**	74	26

**Abbreviations:** HPI = Healthy Places Index; LAC = Los Angeles County; TTS = Test to Stay.

* District student enrollment reported by the California Department of Education as of the 2020–21 school year.

^†^ LAC’s largest school district, which accounts for one third of public-school students, did not adopt TTS.

^§^ Student contacts with unknown school level and age were excluded.

^¶^ Secondary infection risk was defined as the number of quarantined contacts who received a positive SARS-CoV-2 test result 1–14 days after exposure divided by the total number of quarantined contacts. Limited to student school contacts.

** Based on the California HPI, which classifies California zip codes into quartiles based on a composite score of disadvantages. Indicators that determine the HPI score include economic, education, transportation, social, neighborhood, housing, clean environment, and healthcare access. Zip codes falling within the lowest HPI quartile in LAC represent the most disadvantaged neighborhoods. HPI data were missing for 16 non-TTS schools and 22 TTS schools.

Before TTS adoption (August 16–September 19, 2021), average daily student COVID-19 incidence was lower in TTS districts (10 per 100,000 students; 95% CI = 7–13) than in non-TTS districts (20 per 100,000 students; 95% CI = 18–23) (Figure 2). After TTS adoption, average student daily case rates declined in all districts but remained lower on average in TTS districts (6 per 100,000 students; 95% CI = 3–9) compared with non-TTS districts (11 per 100,000 students; 95% CI = 9–13). The ratio of student COVID-19 incidence in TTS districts to that in non-TTS districts was similar before and after TTS adoption (rate ratio = 0.5). (Figure 2).

**FIGURE 2:**
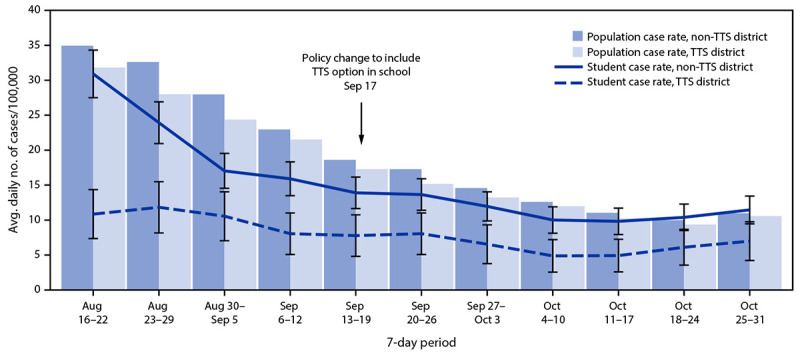
Student and population COVID-19 case rates,* by school district Test to Stay status — Los Angeles County, California, August 16–October 31, 2021 **Abbreviation:** TTS = Test to Stay. * SARS-CoV-2 student case rates were calculated as the average daily number of student cases reported to Los Angeles County Department of Public Health during a 7-day period divided by the number of enrolled students at the school district level. SARS-CoV-2 population case rates were calculated as the average daily number of community cases reported to Los Angeles County Department of Public Health during a 7-day period divided by the population of county residents at the school district level. Standard error bars shown for student case rates.

Among the schools that implemented TTS, 107 of 410 (26%) were categorized as most disadvantaged compared with 1,192 of 1,619 (74%) non-TTS schools.^§§§^ Challenges cited to TTS implementation were limited staffing and systems to monitor mask use, testing, and lack of family support (Supplementary Table, https://stacks.cdc.gov/view/cdc/112641). Non-TTS districts reported similar resource barriers.

## Discussion

Among LAC schools that implemented TTS during September 20–October 31, 2021, COVID-19 incidence did not increase, and tertiary transmission was not identified in school outbreaks after TTS implementation. Non-TTS districts lost substantial in-person school days. Taken together, these findings reinforce the usefulness of TTS for helping to maintain in-person learning in schools.

Only one in five public schools in LAC adopted TTS, and non-TTS schools cited resource-related reasons for opting out of TTS. Inability to implement TTS might exacerbate health and educational disparities between TTS and non-TTS schools. Operationalizing TTS requires staffing resources and systems for monitoring eligibility for and compliance to TTS that might not currently be available in schools in disadvantaged communities, including most LAC non-TTS schools. Moreover, because TTS is currently permitted for quarantined students only during regular school hours, families who rely on before- and after-school programs might opt for home quarantine.

The findings in this report are subject to at least three limitations. First, monitoring systems were not established to assess compliance with TTS requirements or designed to evaluate school transmission before and after TTS adoption. Second, this analysis relied on the existing school case reporting system to characterize school transmission after TTS adoption. Because TTS schools were not required to inform LAC DPH about which students participated in TTS, tertiary transmission from a student participating in TTS could not be determined in non-outbreak settings. Finally, rates were unadjusted and did not control for confounders. However, non-TTS schools were disproportionately located in the most disadvantaged neighborhoods, where population case rates tend to be highest (*7*); this might explain the difference in student case rates in TTS and non-TTS schools.

Preliminary data from LAC suggest that a school-based TTS strategy in a large and diverse county did not increase school transmission risk and might greatly reduce loss of in-person school days. Thus, schools might consider TTS as an option for keeping quarantined students in school to continue in-person learning. However, the resources and operational complexities required to implement school-based TTS might present barriers, particularly for disadvantaged schools. Efforts to better understand barriers and simplify school quarantine strategies might help ensure that all students have access to safe in-person education. Although vaccination remains the leading public health recommendation to protect against COVID-19 for those aged ≥5 years, schools might consider TTS as an option for allowing close contacts who are not fully vaccinated to remain in the classroom as an alternative to home quarantine.

**Summary**

**What is already known about this topic?**

Los Angeles County Department of Public Health permits Test to Stay (TTS) as a COVID-19 quarantine strategy that allows students with school exposures to remain in school if both infected and exposed persons wore masks.

**What is added by this report?**

One in five LAC public schools adopted TTS. In TTS schools, student case rates did not increase, and tertiary transmission was not identified. A higher percentage of disadvantaged schools did not implement TTS.

**What are the implications for public health practice?**

TTS does not appear to increase transmission risk in public schools and might greatly reduce loss of in-person school days. Implementation requires resources that might be currently unavailable for some schools. Vaccination remains the leading recommendation to protect against COVID-19; TTS allows students with a school exposure to remain in the classroom as an alternative to home quarantine.

## Footnotes

* California Department of Public Health’s Test to Stay strategy in k–12 schools is described in section 8 of the COVID-19 Public Health Guidance for K–12 Schools in California, 2021–22 School Year. https://www.cdph.ca.gov/Programs/CID/DCDC/Pages/COVID-19/K-12-Guidance-2021-22-School-Year.aspx

† A school outbreak was defined as three or more epidemiologically linked COVID-19 school-associated cases within 14 days. Outbreaks that occurred after September 20, 2021, were reviewed. Because students participating in TTS were permitted to continue in-person academic activities in school only while under quarantine, outbreak data review was limited to outbreaks that occurred in an academic setting at school (e.g., classroom outbreak). Outbreaks in youth sports settings were excluded.

§ Los Angeles County Public School Districts do not include independent, independent charter, regional occupational programs, or Los Angeles County Special Education. Transitional kindergarten is a public school program serving to bridge preschool and kindergarten.

¶ Fully vaccinated persons with school exposures are exempt from quarantine but are recommended to test 5–7 days after their last exposure date. A person is considered fully vaccinated 2 weeks after receiving 1) the Janssen (Johnson & Johnson) COVID-19 vaccine; 2) the second dose of a Pfizer or Moderna mRNA COVID-19 vaccine; or 3) completing the series of a COVID-19 vaccine that is listed for emergency use by the World Health Organization.

** For school-based TTS, the California Department of Public Health recommends the same testing cadence as in standard quarantine: testing immediately after notification of the exposure and subsequent testing on or after day 5 following the date of last exposure.

†† Contacts who had a positive SARS-CoV-2 test result 1–14 days after exposure reported in IRIS but not identified by schools were also classified as having secondary infections. In order to correct surveillance data reporting ambiguities, contacts with a positive SARS-CoV-2 test result date the same as the quarantine start date were not considered to have secondary infections.

§§ School district student enrollment was reported by the California Department of Education for the 2020–21 school year.

¶¶ California HPI classifies California zip codes into quartiles based on a composite score of disadvantages. Indicators that determine the HPI score include economic, education, transportation, social, neighborhood, housing, clean environment, and health care access. Zip codes falling within the lowest HPI quartile in LAC represent the most disadvantaged neighborhoods.

*** Number of lost school days assumed that the quarantined students were under a 7-day quarantine period, and the student might be released from quarantine after day 7 from the last date of exposure, if the student remained without symptoms and had a negative SARS-CoV-2 viral diagnostic test result from a specimen collected on or after day 5 from the last date of exposure. It is assumed that every 7-day student quarantine resulted in 5 days of missed in-person school days. This represents the lower bound of missed in-person school days because quarantined persons without symptoms and a SARS-CoV-2 viral diagnostic test during quarantine were subject to a 10-day quarantine period.

††† The cities of Long Beach and Pasadena have their own health departments, separate from LAC DPH, and were therefore not included in the analysis.

§§§ HPI data were missing for 16 (1%) of 1,635 non-TTS schools and 22 (5%) of 432 TTS schools.
